# CT gastrography and enterography retrospective analysis: thickened diaphragm crura is a false indication for a gastric fundic tumor

**DOI:** 10.3389/fonc.2024.1414252

**Published:** 2024-10-10

**Authors:** Xin Zhang, Ying Wen, Qi Wang, Manman Chen, Ming Yang, Xiaoyu Han, Wenjuan Tang, Heshui Shi

**Affiliations:** ^1^ Department of Radiology, Wuhan Union Hospital, Tongji Medical College, Huazhong University of Science and Technology, Wuhan, China; ^2^ Department of Radiology, Wuhan Hospital of Traditional Chinese Medicine, Wuhan, China; ^3^ Department of Radiology, Guangdong Provincial People’s Hospital, Guangdong Academy of Medical Sciences, Guangzhou, China

**Keywords:** stomach neoplasms, diaphragm crura, CT enterography, adenocarcinoma, gastrointestinal stromal tumors

## Abstract

**Objectives:**

To mitigate the incidence of misdiagnosis and distinguish between gastric fundic tumors (GFTs) and thickened diaphragmatic crura (TDC).

**Materials and methods:**

Computed tomographic enterography (CTE) data from 3844 cases and computed tomographic gastrography (CTG) data from 4351 cases were retrospectively analyzed. A total of 105 cases were selected and categorized into three groups: 47 cases with TDC examined via CTE, 31 with adenocarcinoma, and 27 with gastrointestinal stromal tumors (GIST) examined via CTG. Inter-group differences in age, sex distribution, mass dimensions, mass-stomach interface (MSI), chief complaints, gastric underlying diseases, and enhancement patterns were analyzed.

**Results:**

The misdiagnosis rate of TDC as a tumor by radiologists is approximately 1.2% (47/3844). Age (*p*<0.05), sex ratio (*p*<0.05), mean mass size (*p*<0.05), chief complaint (*p*<0.05) and mass-stomach interface (MSI, *p*<0.05) were significantly different among patients with GIST, adenocarcinoma and TDC. The contrast enhancement pattern of TDC markedly differed from that observed in adenocarcinoma (*p*<0.05) and GIST (*p*<0.05) patients.

**Conclusions:**

Misdiagnosis of GFTs is occasionally and may be challenging to differentiate from TDC using CTE. To drastically lower the chance of misdiagnosis, this research aimed to assist radiologists in identifying and considering the possibility of TDC.

## 1 Introduction

Gastric tumors are categorized into benign and malignant varieties, with gastric adenocarcinoma accounting for 90–95% of malignant gastric neoplasms (1, 2). Polyps and GISTs represent the most prevalent mesenchymal tumors of the gastrointestinal tract and form the majority of benign tumors (3). Additionally, a multitude of tumorous and non-tumorous lesions demand clinical attention. Early detection of these tumors is pivotal for appropriate therapeutic intervention. Imaging modalities, including computed tomography (CT), magnetic resonance imaging (MRI), endoscopic ultrasound, and esophagogastroduodenoscopy, have witnessed significant advancements, enabling clinicians to identify gastric tumors at an early stage. A substantial proportion of gastric adenocarcinomas(19.9%) (4) and GISTs (82%) (5) originate from the stomach, and the complex anatomical architecture of the gastric fundus presents challenges in distinguishing GFTs (6, 7). Notably, our study has identified that misdiagnoses of TDC as GFT occasionally occur in CTE. Our comprehensive literature review has revealed that the majority of publications addressing misdiagnoses in CTE are predominantly concentrated on conditions such as Crohn’s disease, small bowel neoplasia, and various malabsorptive or vascular disorders (8). The present study aims to elucidate the causes of misdiagnosis of TDC as GFT using CTE and to devise strategies to minimize diagnostic errors.

## 2 Materials and methods

This investigation was sanctioned by our Institutional Review Board, with patient informed consent waived for this retrospective analysis.

### 2.1 Patient enrollment process with inclusion criteria

We conducted a thorough retrospective analysis, examining data from 3,844 cases of CTE and 4,351 cases of CTG. The patient selection for the TDC group was meticulously guided by the following criteria: a. Patients who had undergone CTE and whose stomach cavity was well filled. b. Patients suspected of fundus thickening. c. Patients with no detectable lesions in the fundus by gastroscopy or endoscopic ultrasonography. d. Patients re-evaluated within a 1 to 5-year interval. For the adenocarcinoma and GIST groups, the inclusion criteria were as follows: a. Patients who underwent standard CTG with adequately filled stomach cavities. b. Patients exhibiting thickening of the gastric fundus. c. Patients with histologically confirmed adenocarcinoma and GIST. Then patients were categorized into three distinct groups. The adenocarcinoma group comprised 31 patients, the GIST group included 27 patients, and the TDC group encompassed 47 patients. All CT examinations adhered to the protocols established by our department’s standard operating procedures.

### 2.2 Preparation of CTE

Patients were required to fast for a minimum of 10 hours prior to examination (typically from 10 pm the previous day to 8 am the following day). They were then instructed to consume a 2000 mL mannitol solution (prepared with 300 mL of mannitol) in two sittings. Each patient ingested 1500 mL of the mannitol solution over a 1 to 2-hour period, during which urine and feces were excreted. Subsequently, patients received an intravenous infusion of 20 mg anisodamine (10 mg for adolescents), and the time required to access the indwelling needle was documented. Finally, patients consumed an additional 500 mL of mannitol solution and were instructed to retain their urine. CT scanning was performed 15 to 20 minutes later.

Indications for CTE: a. Melena. b. Unexplained diarrhea and abdominal pain. c. Inflammation and tumors of the small intestine. Contraindications for CTE: a. Gastrointestinal tract perforation. b. Gastrointestinal bleeding.

### 2.3 Preparation of CTG

Patients fasted for at least 6 hours before the examination. Each patient consumed 500–800 mL of tap water.

Indications for CTG: a. Unexplained abdominal pain. b. Inflammation and tumors of the stomach. Contraindications for CTG: a. Gastrointestinal tract perforation. b. Gastrointestinal bleeding.

### 2.4 Scanning techniques

CT scans were performed using a high-resolution CT scanner (Somatom Definition AS+; Siemens, Erlangen, Germany). Scan parameters were set at 120 kV, 100–150 mAs, and a 0.5-second rotation for all three passes. Unenhanced CT scans of the abdomen, from the diaphragmatic domes to the inferior border of the pubic symphysis, were conducted at a 1.5-mm section thickness and 1.5-mm reconstruction interval in CTE. The CTG scanning range extended from the diaphragmatic domes to the lower pole of the kidneys. A 1 mL/kg dose of the iodinated contrast material iopamidol was administered via the antecubital vein at a flow rate of 2.5 mL/sec through a 20-gauge needle. The arterial phase was scanned immediately upon reaching the threshold (100 HU), and the venous phase was initiated after a 25-second delay, with the extension of both phases determined based on unenhanced imaging.

### 2.5 Imaging evaluation

Imaging assessments were conducted by two radiologists with 6 and 12 years of experience, respectively, to reach a consensus. Any disagreements between the radiologists were resolved through discussion until a consensus was reached.

The TDC group was examined using CTE, while the adenocarcinoma and GIST groups were evaluated using CTG. Inter-group differences were analyzed in terms of age, gender, mass size, and mass-stomach interface (MSI), which refers to the angle between the stomach fundus and the mass. The right-side angle was typically selected for evaluation, as the stomach wall was more stretched and relaxed, providing a direct reflection of the mass angle and the natural state of the stomach wall. The two most common GFT and TDC types were subsequently fitted with enhancement curves, and significant differences were analyzed.

### 2.6 Diaphragm crura thickness database of healthy individuals

**Figure 1:**
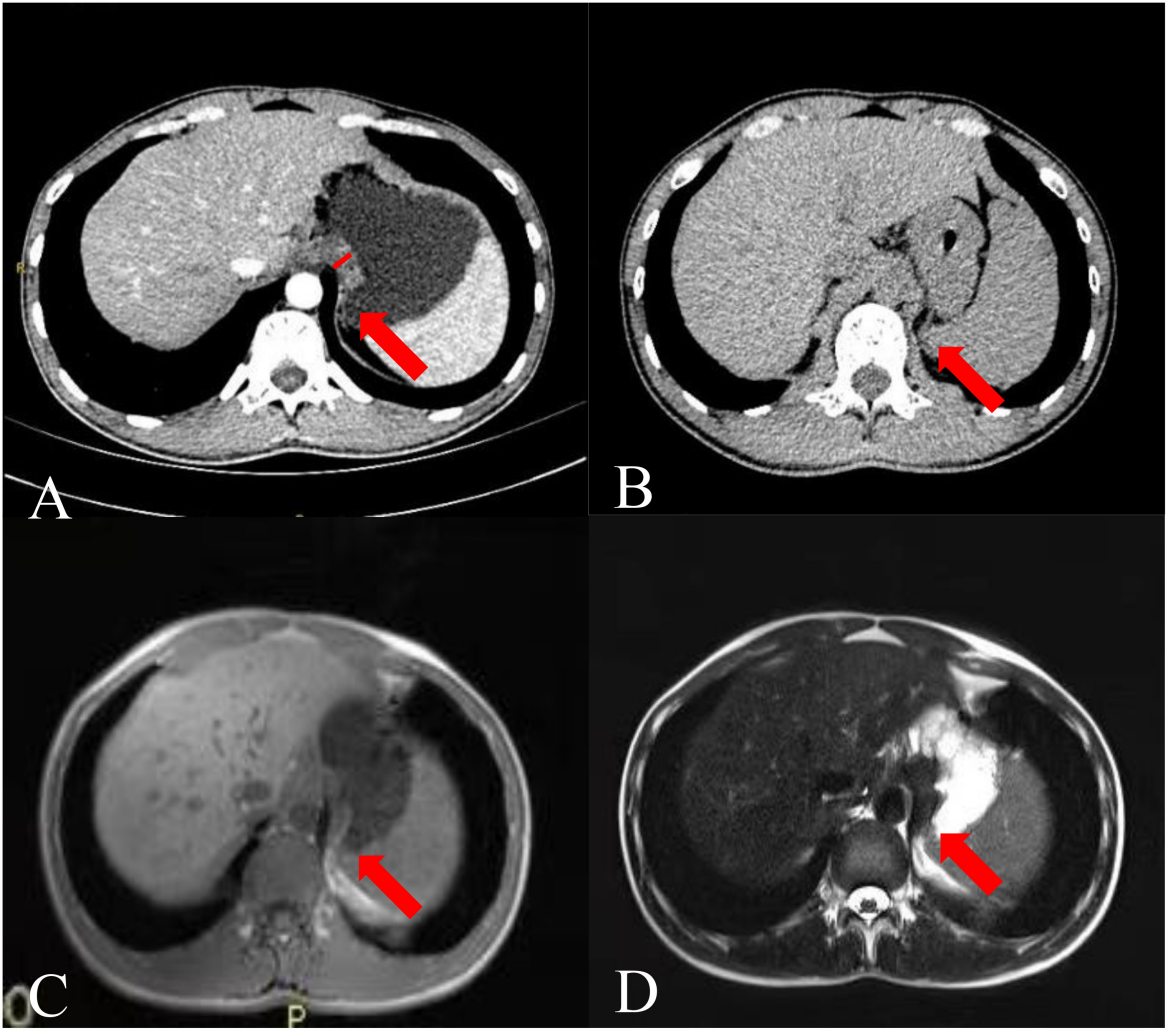
Images of a 26-year-old man. **(A)** CTE image showing thickening of the gastric fundus. The arrow points to the TDC, and the white vertical line indicates the TDC. **(B)** Single-phase upper abdominal CT image showing that the TDC was similar to that of the gastric fundus on CTE. **(C)** Single-phase gastric MR T1 weighted image and **(D)** T2 weighted image showing that the thickened part of the gastric fundus was the impression of the diaphragm crura.

Between May 2016 and March 2023, a subset of CTG and CTE cases was randomly selected by computer to establish a diaphragmatic crura thickness database for healthy individuals. The thickness of the diaphragm crura was measured twice at the level of the cardia (**Figure 1B**) by two radiologists and then averaged.

### 2.7 Statistical analysis

**Figure 2:**
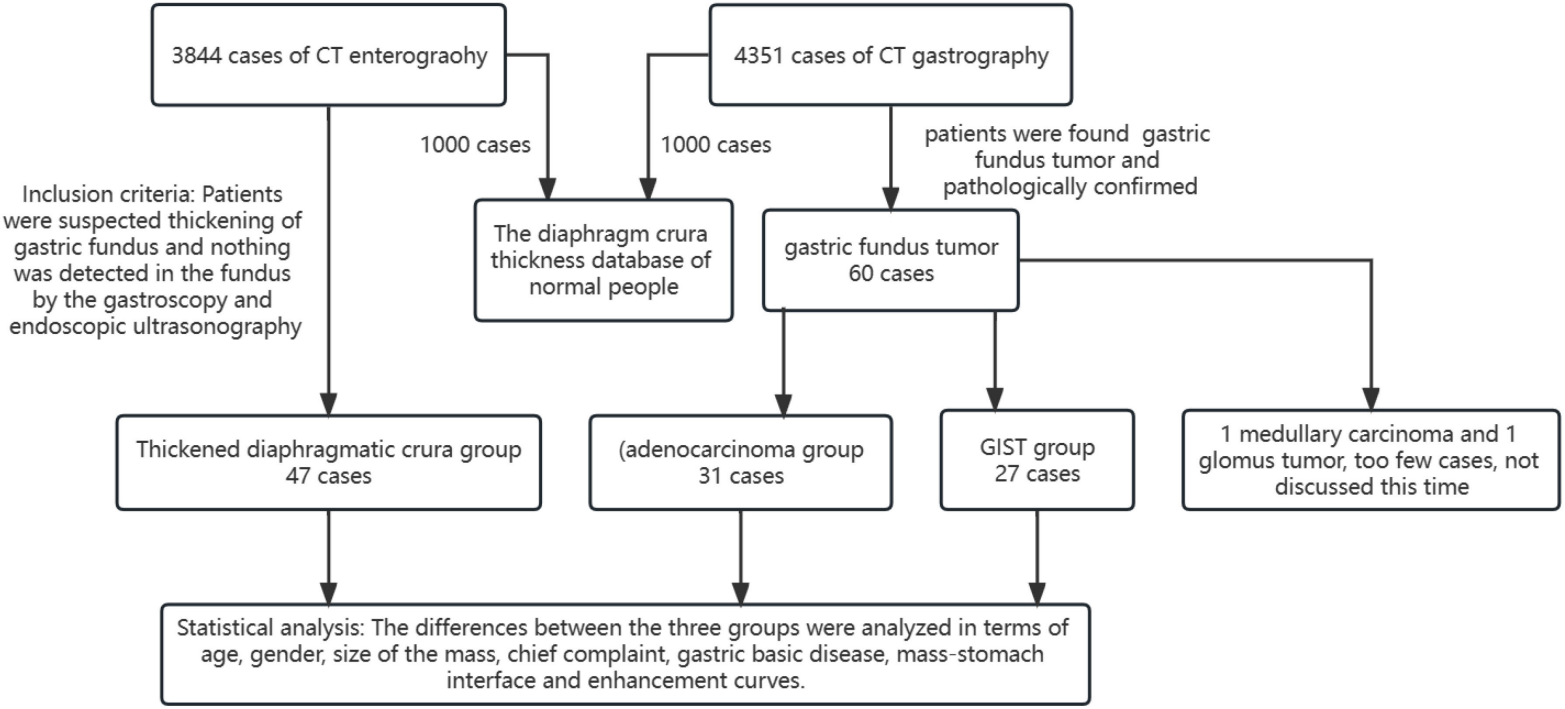
Flow chart of the study.

Statistical analyses were performed using SPSS 23.0 software (SPSS, Inc., Chicago, IL). Numerical data are presented as the mean ± standard deviation. The following statistical tests were employed for data analysis: chi-square test, Fisher’s exact probability, Kolmogorov-Smirnov test, One-way ANOVA test, Kruskal-Wallis test, and regression analysis. A *p*-value of less than 0.05 was considered statistically significant. **Figure 2** illustrates the research process.

## 3 Results

### 3.1 Group situation

Among the 3844 CTE cases, the incidence rate of GFT was approximately 0.2% (8 cases). However, the misdiagnosis rate of TDC as a tumor by radiologists is approximately 1.2% (47 cases). No such misdiagnosis was observed in CTG. Forty-seven cases exhibited localized thickening of the fundus, with an average patient age of 45.8 years, including 21 males (44.7%) and 26 females (55.3%). On CTG, 60 GFT cases were identified among the 4351 patients. Medullary carcinoma and glomus tumors were too infrequent for detailed discussion.

### 3.2 Chief complaint and patient characteristics

In the TDC group identified by CTE, chief complaints included abdominal pain (29 cases), abdominal distention (3 cases), asymptomatic presentation (2 cases), melena (3 cases), constipation (3 cases), diarrhea (4 cases), and weight loss (3 cases). In the CTG-identified GIST group, chief complaints included abdominal pain (9 cases), abdominal distention (3 cases), asymptomatic presentation (12 cases), hematemesis (2 cases), and melena (1 case). In the adenocarcinoma group identified by CTG, chief complaints included abdominal pain (6 cases), abdominal distention (9 cases), asymptomatic presentation (4 cases), hematemesis (1 case), dysphagia (5 cases), melena (2 cases), weight loss (1 case), and chest tightness (2 cases). Chief complaints varied significantly among the three groups (**Table 1**, *p*<0.05). No significant differences were noted in the prevalence of gastric basic diseases among the groups (**Table 1**, *p* =0.14). The symptoms of the three groups exhibited some overlap, with the TDC group primarily characterized by abdominal pain and diarrhea, the GIST group by abdominal pain and asymptomatic presentation, and the adenocarcinoma group by abdominal pain and distention. However, variations in symptoms were observed among the groups, with hematemesis occurring exclusively in the GIST and adenocarcinoma groups, and dysphagia and chest tightness being unique to the adenocarcinoma group.

**Table 1:** Characteristics of patients with GIST, adenocarcinoma and TDC.

Characteristic	GIST	Adenocarcinoma	TDC	*p value*
Mean age ± S.D.(years)	58.5 ± 12.8	65.3 ± 6.6	45.8 ± 12.6	0.004^a^
				0.0004^b^
				<0.001^c^
Sex(M:F)	16:11	25:6	21:26	0.007
Mean mass size ± S.D.(cm²)	11.7 ± 12.9	6.1 ± 4.3	2.8 ± 2.1	0.039^a^
				0.001^b^
				0.0003^c^
Chief complaint				
abdominal distention	3	9	3	<0.001
abdominal pain	9	6	29	
no stomach symptom	12	4	2	
haematemesis	2	1	0	
dysphagia	0	5	0	
melena	1	3	3	
astriction	0	0	3	
diarrhea	0	0	4	
lose weight	0	1	3	
chest tightness	0	2	0	
Gastric basic disease				
nonatrophic gastritis	7	7	6	0.139
atrophic gastritis	1	41	1	
no gastritis or evidence	19	20	40	
Mass-stomach interface				
acute angle	21	3	3	<0.001
approximately right angle	4	3	18	
obtuse angle	2	25	26	

GIST, gastrointestinal stromal tumor; S.D., standard deviation; NS, no significant.

^a^GIST group vs. adenocarcinoma group; ^b^GIST vs. TDC; ^c^Adenocarcinoma group vs. TDC.

The mean ages of patients with GIST and adenocarcinoma, GIST and TDC were found to be similar (**Table 1**). However, a noticeable difference in the mean age was observed between adenocarcinoma and TDC (**Table 1**). Significant differences in sex ratio and mean mass size were noted among GIST, adenocarcinoma, and TDC (**Table 1**, *p*<0.05).

### 3.3 Differences in mass-stomach interfaces

**Figure 3:**
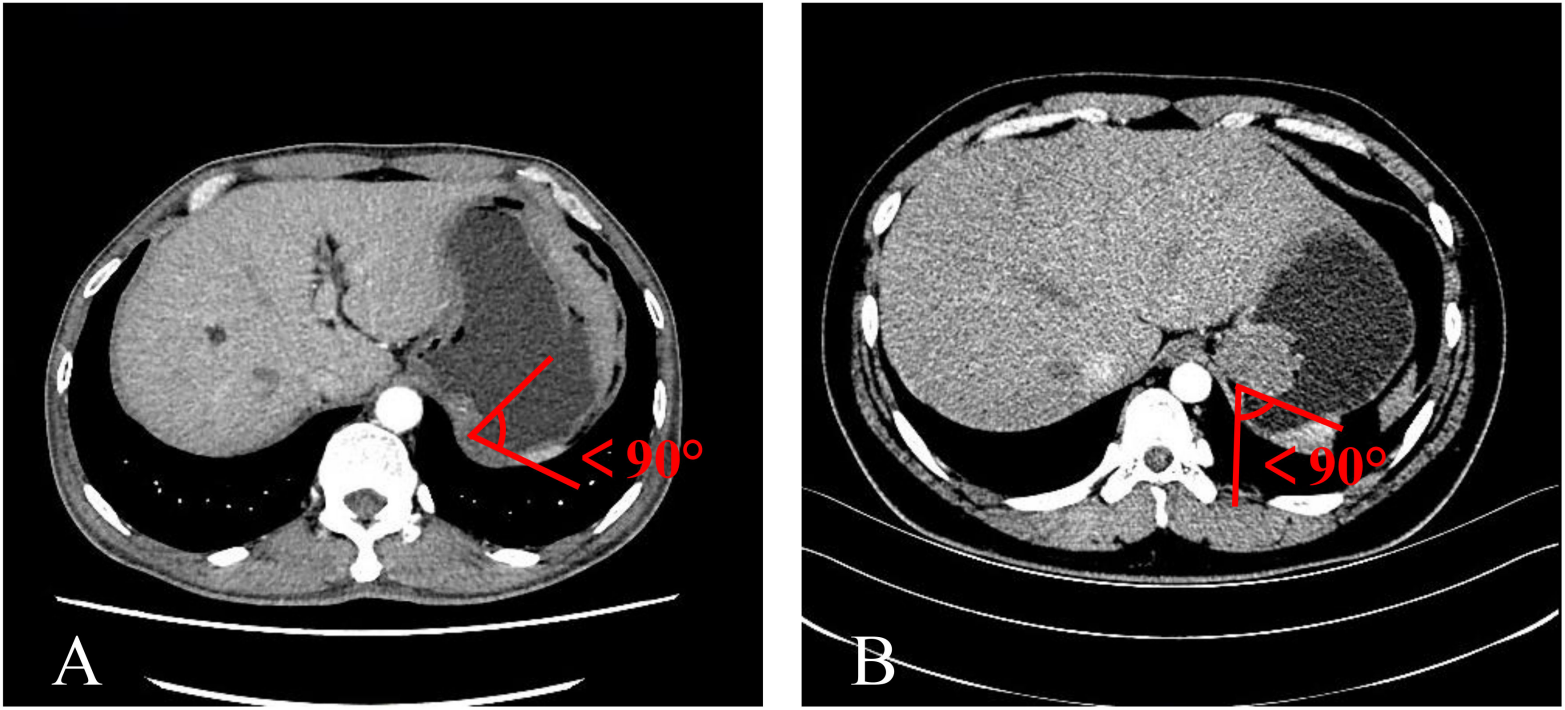
The MSI was an acute angle. **(A)** TDC. **(B)** GIST.

**Figure 4:**
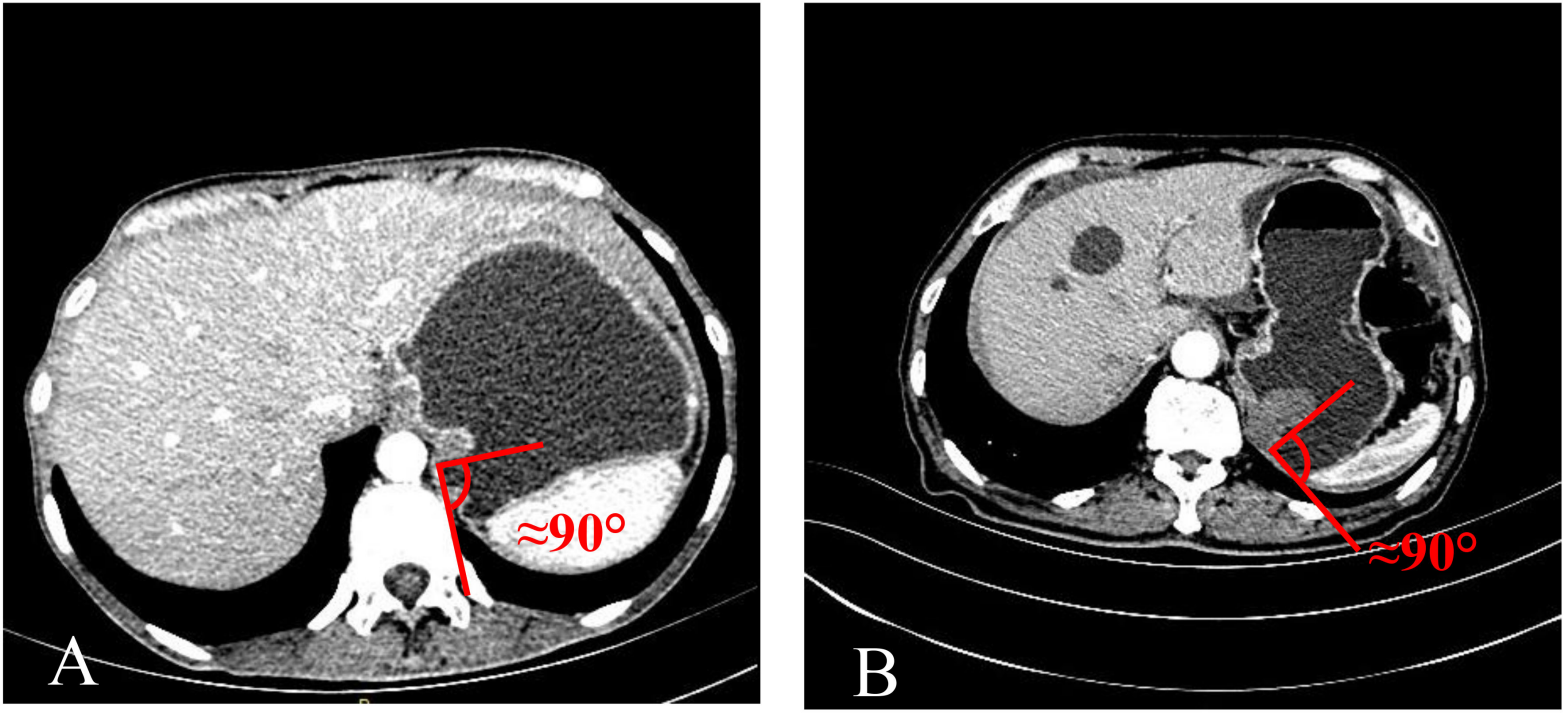
The MSI was approximately right angle. **(A)** TDC. **(B)** GIST.

**Figure 5:**
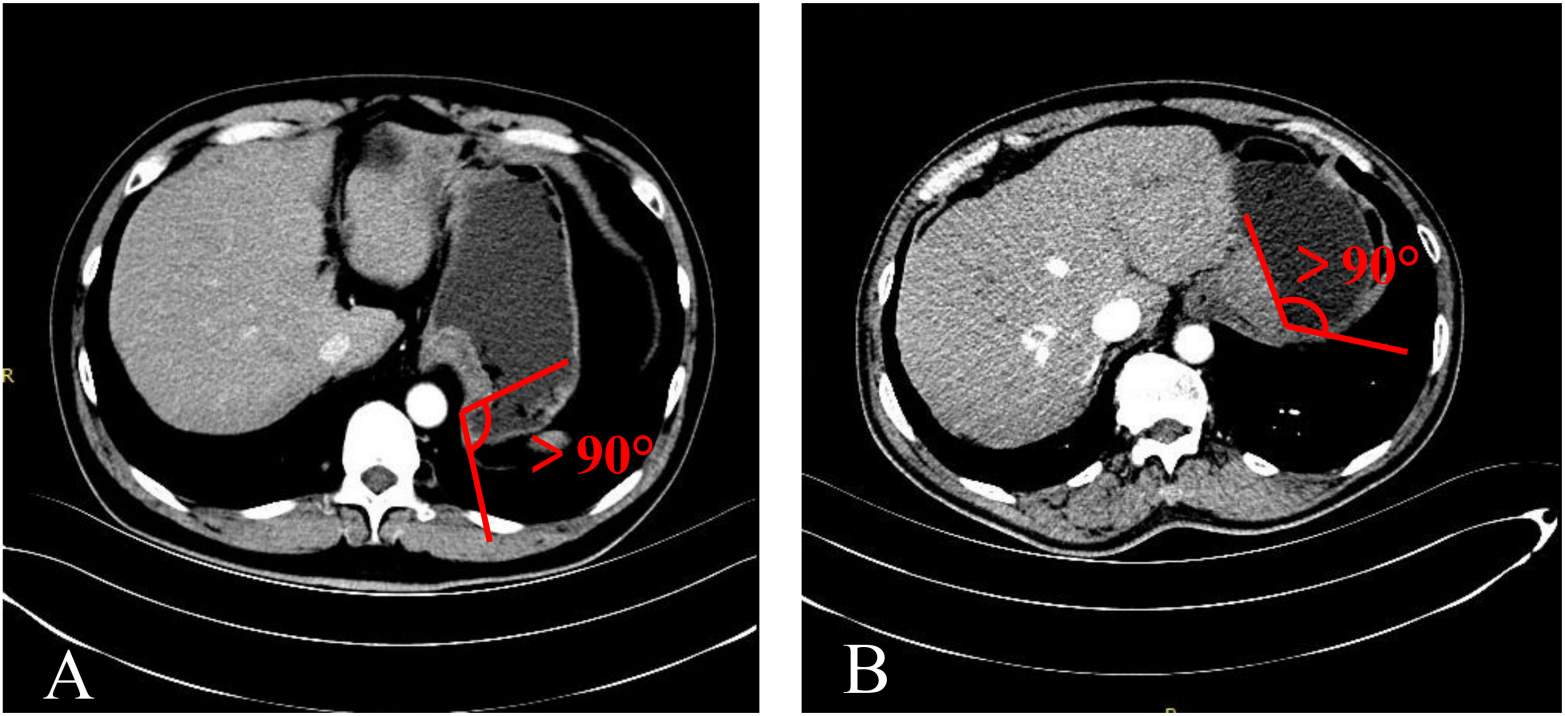
The MSI was an obtuse angle. **(A)** TDC. **(B)** Gastric adenocarcinoma.

The mass-stomach interface (MSI) could present as an acute angle (**Figure 3**), an approximate right angle (**Figure 4**), or an obtuse angle (**Figure 5**). For GISTs, the MSIs were acute in 21 cases, approximately right in 4 cases, and obtuse in 2 cases. Adenocarcinomas exhibited MSIs that were acute in 3 cases, approximately right in 3 cases, and obtuse in 25 cases. TDC MSIs were acute in 3 cases, approximately right in 18 cases, and obtuse in 26 cases. Significant differences were observed among the three groups (*p*<0.05).

### 3.4 Differences in enhancement trends

**Figure 6:**
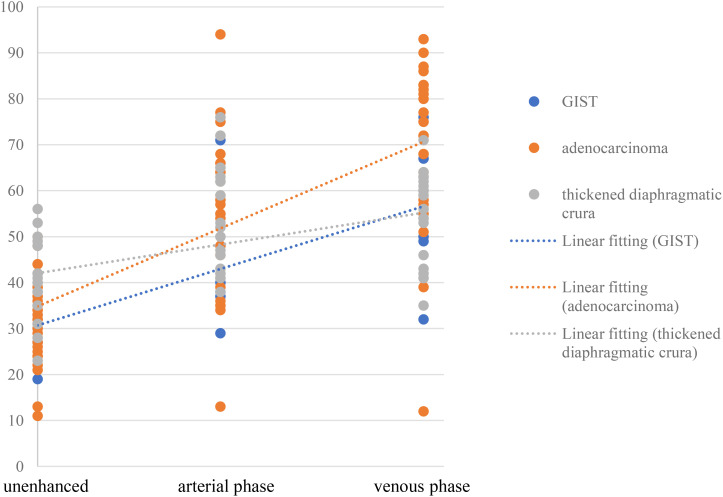
The trend toward tumor enhancement in patients with adenocarcinoma, GIST or TDC.

The contrast enhancement pattern of TDC was significantly distinct from that of adenocarcinoma (*p*<0.05) and GIST (*p*<0.05) (**Figure 6**). The degree of enhancement in TDC was lower than that observed in GFTs, more precisely, the enhancement velocity was slower. No significant difference was found in the enhancement patterns between adenocarcinomas and GISTs (*p* =0.147). However, the absolute CT values of adenocarcinomas were higher than those of GISTs. Additionally, some adenocarcinomas and GISTs exhibited pronounced heterogeneous enhancement, with adenocarcinomas showing a higher prevalence. In contrast, TDC did not display heterogeneous enhancement, thus yielding no relevant statistical evidence.

Adenocarcinoma, GIST, and TDC shared some common features. For instance, thickening of the stomach wall with soft tissue density and homogeneous enhancement could occur when the base was wide or narrow. However, they also exhibited distinct characteristics. The detection of adenocarcinoma and GIST was based on the presence of abnormalities and enhancement in the stomach wall, typically characterized by irregular thickening and uneven enhancement.

### 3.5 Diaphragmatic crura thickness on CT images

A random selection of 1000 CTG cases (out of a total of 4351) and 1000 CTE cases (out of a total of 3844) was used to establish a database for the thickness of the diaphragmatic crura in healthy individuals (976 males and 1024 females; age range, 9-90 years old; mean age, 50 years old). The data distribution was right-skewed. The average thickness of the diaphragmatic crura in this database was approximately 4.7 mm, with a standard deviation of 2.1 mm. Over 95% of the diaphragmatic crura measured less than 8.4 mm in thickness. Among the 47 cases of TDC, the thickest measured 19.6 mm, and the thinnest was 8.5 mm. There was no significant difference between CTG and CTE (*p*=0.233). Significant differences in the thickness of the diaphragmatic crura were observed between different age groups (*p*<0.05).

## 4 Discussion

### 4.1 Reasons for misdiagnosis of TDC as GFT in CTE

The misdiagnosis rate of TDC as a tumor in CTE was approximately 1.2%. In contrast, the incidence rate of GFT in CTE was a mere 0.2%. Notably, the misdiagnosis rate of gastric tumors in CTE was nearly six times the actual diagnostic rate. Intriguingly, no similar misdiagnoses were encountered in CTG.

Due to the similarities in morphology, enhancement pattern, and clinical symptoms, coupled with a lack of clinical attention to the diaphragmatic crura’s structure, misdiagnosis was not uncommon. CT scans performed near the diaphragm may reveal the crura differently as fibers extend from the vertebral bodies toward the aorta. This can complicate the identification of correct anatomy, particularly when sections are slightly caudal and only partially display the crus. Additionally, structures adjacent to the crura (depending on the level of the section) might be confused with other diseases or diagnoses (6, 7). Our study found that the thickness of the diaphragmatic crura in 95% of healthy individuals was less than 8.5 mm. Lesions equal to or greater than 8.5 mm in length might exhibit a locally thickened appearance, resembling the appearance of GFT in CTE. During examination, patients are required to take deep breaths and hold their breath, which can induce nodular changes in the diaphragmatic crura (9). Moreover, during early enhancement, the gastric mucosa was significantly enhanced, while the diaphragmatic crura remained relatively weak, creating a false image of uneven enhancement akin to that of a GFT. The overlap in symptoms (primarily abdominal pain and distension) with those of GFT (10) further escalated the misdiagnosis rate.

The key sign of TDC misdiagnosed as a GFT was the disappearance of the fatty space between the stomach fundus and the diaphragm crura in CTE. Anisodamine reduces gastric tension (11), leading to an increased contact surface between the gastric wall and the dorsal abdominal wall, particularly in the area near the diaphragmatic crura. Additionally, with the patient in a supine position during the examination and the stomach well-filled and shifted backward under the influence of gravity, the fatty space between the stomach and the diaphragmatic crura disappears, leading to the false appearance of a mass at the stomach fundus (**Figure 1B**). However, an abdominal plain CT scan can reveal the fatty space between the stomach fundus and the diaphragm crus (**Figure 1C**), aiding in differentiating between the two structures. The fatty space between the fundus and the diaphragmatic crus was typically not visible, complicating diagnosis and potentially leading to misdiagnosis.

### 4.2 Analysis of differences between TDC and GFT

Beyond individual variability, our research indicates that age plays a role in the morphology of the diaphragmatic crura. Our data from the general population revealed a statistically significant difference in the thickness of the diaphragm crura across different age groups (*p*<0.0001). Literature suggests that relative to body size, the diaphragmatic crura are largest at birth and gradually decrease with age (12). Similarly, a nodular contour to the diaphragm is common in infants and less so with increasing age through childhood. The crus becomes thinner and less nodular with aging and expiration (9). Our findings indicate that the average age of patients misdiagnosed with TDC (45.8 years old) was younger than that of tumor patients (59.3 or 65.3 years old). There were 6 cases in the TDC group under the age of 30. Therefore, if a patient is younger, the possibility that the suspected lesion of the gastric fundus is TDC must be considered in CTE. TDC can occur in younger individuals, potentially due to age-related loss of skeletal muscle mass (13). Although TDC is more common in young people, the trend of tumor rejuvenation also complicates differentiation.

This study found no significant difference (21:26) in the incidence of TDC between males and females. However, adenocarcinoma (25:6) and GIST (16:11) predominantly occurred in males. Other studies suggest that adenocarcinoma is more prevalent in men (14), while the incidence of GIST is similar between males and females (15). Gender differentiation offers limited assistance in our identification process.

The TDC was significantly smaller in these patients than in those with GISTs (**Table 1**). Sandrasegaran et al. (16) reported that the diameter of GISTs usually ranges between 3 to 10 cm, which aligns with our findings. The primary difference in the area of TDC and GFT may be attributed to the biological characteristics of the tumor, which tends to result in uncontrolled dysplasia. While related studies did not separately consider the area or size of adenocarcinoma lesions, the differences in size observed in our study hold diagnostic value.

Our research uncovered significant differences among these three groups (**Table 1**). In terms of morphology, TDC could be categorized into two types: the protruded type (**Figures 3A**, **4A**) and the flat type (**Figure 5A**). The distinction between them lies in the MSI being less than or equal to 90°for the former and greater than 90° for the latter. We found that the MSI of most GISTs was less than 90°, and 80.6% of adenocarcinomas were greater than 90°. Thus, differentiating protruded TDCs from GISTs and flat TDCs from adenocarcinomas is of heightened importance. The TDC, located outside the stomach wall, may be right or obtuse due to factors such as its inherent shape, high pressure on the stomach wall, and depth of respiration. Adenocarcinoma, which arises from the gastric mucosa and submucosal tissues without a capsule or surrounding tissues (17), exhibits an infiltrative growth pattern and varied shapes. The MSI of adenocarcinoma is predominantly obtuse. GISTs originate from the muscles of the stomach, with peripheral muscular, mucosal, and serosal layers on both sides, demonstrating expansive growth, and the MSI typically reveals an acute angle. In clinical practice, the diagnostic value of MSI is limited.

TDC, adenocarcinoma, and GIST exhibit different enhancement modalities and levels due to their pathological underpinnings and distinct biological behaviors. Pathologically, TDC is composed of skeletal muscle tissue, which lacks tumor angiogenesis compared to the former two, and its enhancement is more homogeneous, with a slowly increasing enhancement trend. Adenocarcinoma, occurring in the mucosal layer, can destroy the mucosal layer and promote the proliferation of connective tissue in the submucosa (18), while GIST arises from Cajal cells in the gastric muscle wall and is primarily composed of spindle-forming fibroblasts, epithelioid cell proliferation, a compact structure, and relatively few blood vessels (19). Consequently, the degree of enhancement in adenocarcinoma surpasses that in GIST, and the enhancement period occurs slightly earlier than that in GIST. Compared with GIST and adenocarcinoma, TDC is not associated with tumor angiogenesis, the degree of enhancement is more homogeneous, and the degree of enhancement increases slowly (**Figure 6**). If a mass in the gastric fundus is suspected to be due to TDC, it is crucial to measure the CT value of the mass and the opposite side of the diaphragmatic crus in each phase. This is because the enhancement of both sides of the diaphragmatic crus is consistent.

### 4.3 Preliminary diagnostic strategy

In conjunction with the findings, when diagnosing a GFT in CTE, it is imperative to check for the presence of fatty space between the stomach wall and diaphragmatic crura. If detected, it is more likely a GFT. In the absence of such space, TDC should be suspected. Secondly, measuring the CT values of the suspicious lesions and the contralateral diaphragmatic crus at each stage can provide further insight. Consistency in CT values suggests a higher probability of TDC, while inconsistency points towards a GFT or other possibilities. Moreover, if the patient is younger or presents with a smaller mass, the likelihood of diaphragmatic crura thickening is greater; otherwise, the likelihood of a GFT is higher.

After analyzing the current issues, we propose several strategies to aid in distinguishing between potential diagnoses. We recommend re-examining a CT scan of the upper abdomen within 1 to 3 months. At this juncture, separation between the gastric fundus and the diaphragm crus is often observed (**Figure 1C**). If the sensation is detected promptly, we advocate capturing a prone position image immediately and instructing the patient to hold their breath naturally rather than inhaling deeply. We speculate that the separation of the gastric fundus and diaphragmatic crus may be attributed to changes in gravity, abdominal pressure, and respiration, which may assist in distinguishing GFT from TDC. After obtaining consent from one patient suspected of having TDC, an upper abdominal plain MRI was performed for verification purposes and not included in the main part of our study. Gastroptosis and low gastric tension may elucidate the confusion surrounding TDC in the absence of antispasmodics. The MSI of TDC was similar to that of adenocarcinoma, but it appeared isointense on both T1 and T2 weighted images (**Figures 1D, E**). Conversely, adenocarcinoma typically presents as slightly hyperintense on T1 and T2 (14), facilitating differentiation from the TDC. Although the patient’s MR signal parallels that of many GISTs (15), the MSI of GISTs is acute. In this scenario, we can distinguish it from the two most common GFTs. However, based on the information provided, TDC can assume various shapes, complicating differentiation.

### 4.4 The benefits and limitations of research

In CTE, identifying suspicious fundal lesions is encouraged to improve health outcomes. However, false diagnoses can lead to unintended harm, including extended lead times, heightened health anxiety, increased risk compensation, rebound effects, guilt, and stigma (20). Such harm may also manifest as unnecessary and ineffective invasive procedures, such as gastroscopy.

Nevertheless, our research has its limitations. We selected patients with CTG and CTE for comparison, which may not be methodologically rigorous. However, this was a retrospective study. We initially identified the false appearance of GFT on CTE and then considered whether this situation would be present in CTG. Research has shown significant differences between the two. We further explored the causes of these differences and conducted a retrospective study. The technical parameters of both are identical. The only difference lies in the use of antispasmodic drugs and the dosage of negative contrast agents. Additionally, the measurement of diaphragmatic crura is based on data from a single center, including pediatric data. This may affect the thickness standard of diaphragmatic crura in the general population. Looking ahead, we are contemplating the execution of a multicenter survey to assess the prevalence and characteristics of the diaphragmatic crus in the general population. This initiative aims to delve deeper into the morphology and anatomical variations of the diaphragmatic crus. Such research endeavors will be instrumental in enhancing diagnostic accuracy for gastric fundus conditions, thereby offering valuable insights to clinicians and researchers alike.

## 5 Conclusions

In CTE, differentiating between localized fundal thickening due to TDC and neoplasia is crucial. In the past, the presence of TDC has often been overlooked, leading to misdiagnoses of TDC as GFTs in CTE. To reduce the rate of misdiagnosis, it is essential to examine the fat space between the diaphragmatic crus and the gastric fundus, and to compare the suspected lesion’s enhancement with that of the opposite diaphragmatic crus before establishing a diagnosis. If the diagnosis remains ambiguous, a follow-up abdominal CT scan can be performed 1 to 3 months later. This study aims to assist radiologists in recognizing the presence of TDC and considering this possibility, thereby effectively reducing the likelihood of misdiagnosis and alleviating the psychological and economic burden on patients.

## Data Availability

The raw data supporting the conclusions of this article will be made available by the authors, without undue reservation.
